# Optimization of phenolic compound extraction from Tunisian squash by-products: A sustainable approach for antioxidant and antibacterial applications

**DOI:** 10.1515/biol-2025-1096

**Published:** 2025-04-28

**Authors:** Walid Yeddes, Feten Zar Kalai, Iness Bettaieb Rebey, Majdi Hammami, Neji Tarchoun, Lilian Barros, Spyridon A. Petropoulos, Hanen Falleh, Riadh Ksouri

**Affiliations:** Laboratory of Aromatic and Medicinal Plants, Center of Biotechnology of Borj Cedria, BP 901, 2050, Hammam-Lif, Tunisia; Institut Supérieur Agronomique de Chott Mariem, B.P 47, 4042, Chott Mériem Sousse, Tunisia; Centro de Investigação de Montanha (CIMO) Instituto Politécnico de Bragança, Campus de Santa Apolónia, 5300-253, Bragança, Portugal; Laboratório para a Sustentabilidade e Tecnologia em Regiões de Montanha, Instituto Politécnico de Bragança, Campus de Santa Apolónia, 5300-253, Bragança, Portugal; University of Thessaly, Department of Agriculture, Crop Production and Rural Environment, Fytokou Street, 38446, Volos, Greece

**Keywords:** antimicrobial activity, antioxidant activity, *Cucurbita maxima*, squash by-products, sustainable extraction, waste valorization

## Abstract

The valorization of agricultural by-products is a key strategy for environmental sustainability. This study focuses on optimizing the extraction of phenolic compounds from by-products (peels, fibrous strands, and seeds) of two Tunisian squash landraces (e.g. Bejaoui and Karkoubi) using the response surface methodology to enhance their antioxidant and antibacterial properties. Ethanol concentration, extraction time, and temperature were the key parameters evaluated for their impact on phenolic compounds yield and bioactivity. High-performance liquid chromatography identified the major bioactive phenolic compounds, including vanillic acid, catechin gallate, hydroxytyrosol, and chlorogenic acid. The optimal extraction conditions for each by-product were defined as follows: Bejaoui peels (51.5% ethanol, 40.8°C, 50.5 min), fibrous strands (50.4% ethanol, 37.1°C, 36.3 min), and seeds (30% ethanol, 36.4°C, 8 min); Karkoubi peels (13.2% ethanol, 43.4°C, 47.2 min), fibrous strands (33.4% ethanol, 46.6°C, 10.8 min), and seeds (10.65% ethanol, 55.34°C, 23.16 min). The results demonstrated that optimizing extraction conditions may lead to significant enhancement of the total phenolic content and antiradical activity, with experimental values closely matching predictive models. Furthermore, the bioactive properties of these by-products, particularly their antibacterial activity, highlight their potential application as novel eco-friendly matrices for natural antioxidant and antimicrobial agents. This study underscores the importance of optimizing sustainable extraction techniques to maximize the valorization of agricultural waste, contributing to both environmental protection and the development of innovative natural products within the circular economy concept.

## Graphical abstract

**Figure:**
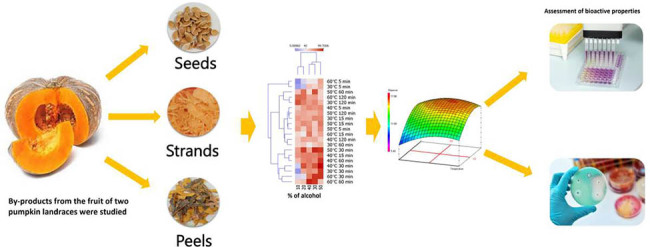

## 1 Introduction

Optimizing agricultural by-products’ utilization is an imperative task to add value to these by-products [1,2]. Antioxidants are substances that prevent or delay the oxidation of other molecules by inhibiting the onset or progress of oxidation chain reactions. They are commonly added to food products, particularly those containing lipids, to extend their shelf-life by slowing down the process of lipid peroxidation, which is a major cause of food spoilage during processing and storage [3]. Antioxidants can be either synthetic or naturally occurring, although the use of the former is gradually being limited because they are potentially carcinogenic Scientific research revealed that commonly utilized synthetic antioxidants like butylated hydroxytoluene, butylated hydroxyanisole, and tertiary butyl hydroquinone have harmful impacts on human health and are often associated with carcinogenesis [4]. Currently, there is a rising trend of utilizing natural antioxidants, particularly those derived from plant matrices, since consumers are becoming more concerned about food safety and advantageous health impacts of consuming fruits and vegetables and functional food products [5,6].

Squash (*Cucurbita* sp.), which belongs to the Cucurbitaceae family, includes annual species that are widely grown around the world and have been used historically as both human food and animal feed. They are cultivated from Northern Mexico to Argentina and Chile and have been spread to Europe (France and Portugal), Asia (India and China), and Western America. Production statistics reveal that India is one of the world’s leading producers of squash [7]. The immature squash fruit can be cooked and consumed as a vegetable, while the mature fruit is sweet and commonly used in pastry making and beverages [8]. However, it has been observed that food processing usually only utilizes the pulp of the squash fruit; thus, approximately 18–21% of waste is produced [9].

The properties of plant extracts rely on various factors such as extraction protocol, solvent type, temperature, pH, extraction steps, liquid-to-solid ratio, and solute particle size that may affect extraction yield and chemical composition [10,11]. To ensure efficient extraction of the target compounds without any chemical alteration, appropriate extraction parameters must be selected [6,12]. The extraction technique utilized depends on the chemical properties and distribution of phenolic compounds in the plant matrix [13]. Due to the nature of phenolic compounds, different solvents and procedures are used for extraction purposes. Ethanol, which is a polar solvent, is frequently used to extract polyphenols from different plant matrices [14]. However, a single solvent may not be adequate to efficiently extract phenolic compounds from most of plant materials, and combinations of aqueous-ethanolic solvents are usually more efficient in recovering antioxidants than pure solvents [15]. In classical optimization experiments, only a single factor is variable at a time, and this method is called the one-factor-at-a-time approach. However, this technique is tedious, expensive, time-consuming, and fails to reveal the interaction effects between extraction variables. Conversely, response surface methodology (RSM) is a useful method to evaluate the effects of multiple factors and their interactions on one or more response variables at the same time [16,17]. RSM can effectively be used to find a combination of factor levels that result in the optimum response [12,18–20]. One of the main advantages of this method is that it generally requires fewer experimental runs compared to full factorial designs, while providing statistically acceptable results.

The objective of this study was to use the RSM approach to optimize the extraction conditions (e.g., solvent, extraction temperature, and extraction time) of bioactive compounds from squash by-product and to assess the total phenolic compound content, free radical scavenging capacity, and antibacterial activity of the obtained extracts. The obtained results could be useful to the food and nutraceutical industry through the application of novel natural compounds as substitutes for synthetic antimicrobial agents.

## 2 Experimental

### 2.1 Plant material

In the present study, two widely cultivated squash (*Cucurbita maxima* Duchesne) were tested, namely Karkoubi (NGB748) and Bejaoui (NGB751). The two landraces were assigned passport data and an inventory number, according to the National Gene Bank of Tunisia, while full details are available at the Germplasm Resources Information Network (GRIN; http://www.tn-grin.nat.tn/gringlobal/search.aspx, accessed on 15 February 2022). Five mature fruits with an average weight of 6 kg were selected. The fruit by-products were manually separated into seeds, fibrous strands, and peels. These were lyophilized using an Alpha 1-2 LD plus lyophilizer from Martin Christ in Osterode, Germany, and the obtained products were ground and stored at −20°C for further analysis.

### 2.2 Preliminary study design

The powdered squash by-products (1 g/10 mL) were subjected to maceration under varying conditions to optimize extraction. Maceration was performed at temperatures of 30, 40, 50, and 60°C, with ethanol concentrations in the solvent (v/v in water) of 10, 20, 30, 40, and 50%. The extraction durations were set at 5, 15, 30, 60, and 120 min. The resulting extracts were concentrated by evaporating the ethanol fraction under reduced pressure using a rotary evaporator, followed by lyophilization to obtain a dry powder.

### 2.3 Analysis of total phenolic compounds content (TPC) and antioxidant activity

The TPC of the powdered squash by-products was determined using the Folin-Ciocalteu assay, as previously described by Zar Kalai et al. [18], and expressed as mg gallic acid equivalents per gram dry weight (mg GAE/g DW). The antioxidant activity was measured using the 2-diphenyl-1-picrylhydrazyl (DPPH^•^) radical scavenging assay, following the method outlined by Zar Kalai et al. [18], and the results were presented as inhibition percentage (IP). All analyses were performed in triplicate.

### 2.4 Extraction through D-optimal RSM

In this study, the D-optimal RSM was utilized to enhance the antioxidant extraction parameters from the tested squash by-products. Specifically, we investigated the influence of three independent variables, ethanol concentration (%), extraction temperature (°C), and extraction time (min), on the targeted responses such as TPC and DPPH free radical scavenging activity (IP), using the Box-Behnken design. A preliminary assessment of the extraction variables was conducted using a single-factor test to determine the independent factors and their values. The experimental design consisted of 17 points, including five replications of the centroids (Table 1). The results were analyzed by fitting them to a second-order polynomial model corresponding to the following equation:

(1) \[Y={b}_{0}+{b}_{1}\times {X}_{1}+{b}_{2}\times {X}_{2}+{b}_{3}\times {X}_{3}+{b}_{11}\times ({X}_{1}\times {X}_{1})+{b}_{22}\times ({X}_{2}\times {X}_{2})+{b}_{33}\times ({X}_{3}\times {X}_{3})+{b}_{12}\times ({X}_{1}\times {X}_{2})+{b}_{13}\times ({X}_{1}\times {X}_{3})+{b}_{23}\times ({X}_{2}\times {X}_{3}),]\]

where *Y* represents the predicted response and *b*_0_, *b*_1_, *b*_2_, *b*_11_, *b*_22_, *b*_33_, *b*_12_, *b*_13_, and *b*_23_ represent the constant, linear, quadratic, and interactive coefficients. The coded levels of the independent variables were represented by *X*_1_, *X*_2_, and *X*_3_, corresponding to ethanol concentration (%), extraction temperature (°C), and extraction time (min), respectively.

**Table 1:** Experimental conditions of the studied plant matrices

*N*° Exp	*X*_1_: % Ethanol	*X*_2_: Temperature (°C)	*X*_3_: Time (min)
1	5.00	50.00	22.50
2	15.00	50.00	22.50
3	5.00	60.00	22.50
4	15.00	60.00	22.50
5	5.00	55.00	15.00
6	15.00	55.00	15.00
7	5.00	55.00	30.00
8	15.00	55.00	30.00
9	10.00	50.00	15.00
10	10.00	60.00	15.00
11	10.00	50.00	30.00
12	10.00	60.00	30.00
13	10.00	55.00	22.50
14	10.00	55.00	22.50
15	10.00	55.00	22.50
16	10.00	55.00	22.50
17	10.00	55.00	22.50

### 2.5 Quantification of squash by-product phenolic compounds by RP-HPLC/diode array detector (DAD)

The phenolic compounds were characterized using an HPLC system (Agilent Technologies 1260 Infinity LC system, Santa Clara, CA, USA) equipped with a reverse-phase C18 column (4.6 mm × 100 mm and 3.5 µm particle size; Zorbax Eclipse XD B C18; Agilent Technologies 1260, Santa Clara, CA, USA). The DAD was set to scan in the range of 200–400 nm. The column temperature was maintained at 25°C. A 2 μL volume of the extract was injected, and the mobile phase flow rate was set to 0.4 mL/min. Mobile phase B consisted of HPLC water with 0.1% formic acid, while mobile phase A was methanol. The optimized chromatographic conditions were as follows: 0–5 min: 10% A–90% B; 5–10 min: 20% A–80% B; 10–30 min: 30% A–70% B; 30–40 min: 50% A–50% B; 40–45 min: 60% A–40% B; 45–50 min: 70% A–30% B; 50–55 min: 90% A–10% B; 55–60 min: 50% A–50% B; and 60 min: 10% A–90% B. The phenolic compounds were identified by comparing their retention times and UV spectra with the corresponding commercial standard.

### 2.6 Antibacterial assay

#### 2.6.1 Bacterial strains

The antibacterial activity was tested against two Gram-positive bacteria (*Enterococcus faecalis* and *Bacillus subtilis*) as well as against three Gram-negative bacteria (*Salmonella enterica*, *Pseudomonas aeruginosa,* and *Escherichia coli*). Each bacterial strain was obtained from American type culture collection.

#### 2.6.2 Antibacterial bioassay

Prior to conducting the antibacterial tests, the bacterial strains were cultivated in liquid nutrient broth (MERCK, Germany) at the specific optimal temperature for each strain for 24 h. The antibacterial properties of peel, strands, and seed extracts from the two pumpkin varieties (Bejaoui and Karkoubi) were investigated using the microplate bioassay [21]. A 100 µg aliquot of dry extracts (DEs) was introduced into sterilized 96-well plates (Fisher Bioblock). After the complete evaporation of the solvent, each well received 100 µL of bacterial suspensions (10^2^ cells/mL) that were prepared by dilution from the culture tubes (10^8^ cells/mL). The bacterial suspension was used without the addition of antibiotics as a positive control or with the presence of an antibiotic mixture (5 mg/mL streptomycin and 10 mg/mL penicillin G) as a negative control (Penicillin-Streptomycin P4458-Sigma-Aldrich). Finally, the microplate was aseptically sealed, stirred, and incubated at the specific optimal temperature for each strain for 24 h. Then, bacterial growth was estimated by reading the absorbance at 405 nm with a microplate spectrophotometer (EZ Read 2000, Biochrom, Cambridge, UK).

#### 2.6.3 Antibacterial activity assessment

The antibacterial activity of peels, strands, and seeds extract obtained from the tested Squash landraces was expressed based on the protocol previously described by Falleh et al. [16] as a percentage of growth inhibition (inhibited by extract). The absorbance data allowed calculating the percentage of growth inhibition using the following formula:

(2) \[\text{Growth inhibition}( \% )=100-(100\times ({A}_{{\mathrm{sample}}}-{A}_{{\mathrm{SC}}})\hspace{10.5em}/({A}_{{\mathrm{GC}}}-{A}_{{\mathrm{SC}}})),]\]

were *A*_sample_ is the absorbance of the sample, *A*_SC_ is the absorbance of the sterility control (negative control, which contains microorganisms without any treatment to ensure no contamination), and *A*_GC_ is the absorbance of the growth control (positive control, which contains microorganisms with the antibiotic, showing inhibition of bacterial growth and confirming the effectiveness of the antibiotic. These controls are essential for accurately comparing the antibacterial activity of the extracts).

### 2.7 Statistical analysis

Heatmap clustering was carried out using Orange Software (version 3.4.5, University of Ljubljana, Slovenia), while the experimental design and statistical analysis were performed using the NemrodW program (version 2000, LPRAI, Marseille, France). Significant differences between the means of independent variables were examined with the analysis of variance (ANOVA) using IBM SPSS Statistics Software (Version 20.0, IBM SPSS Inc., Armonk, NY, USA), followed by means comparison using Duncan’s multiple range test at *p* < 0.05 to avoid any bias due to type I error. Moreover, a regression analysis was also conducted using the abovementioned software to estimate the relationships between the dependent and independent variables. The *R*-squared (*R* ^2^) is a statistical measure that represents the proportion of the variance in the dependent variable that is explained by the independent variable(s). This parameter may range from 0 to 1, with a higher value indicating a better fit of the model to the data.

The adjusted *R*-squared (*R* ^2^_Adj_) is a modified version of *R* ^2^ that takes into account the number of independent variables in the model. It is adjusted for degrees of freedom and penalizes the addition of independent variables that do not improve the fitness of the model.

## 3 Results

### 3.1 Preliminary assessment for maceration processing

**Figure 1:**
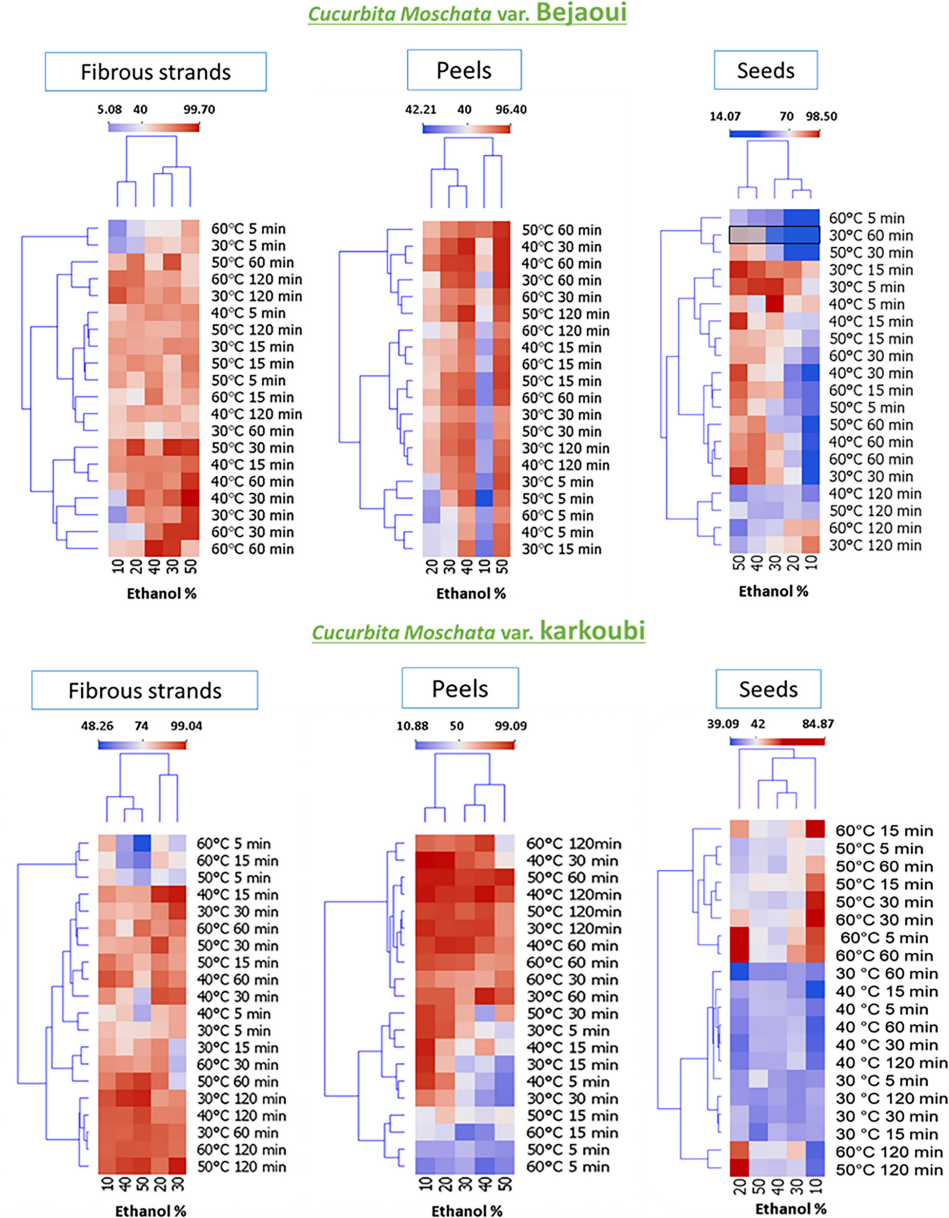
Heatmap clustering presenting the effects of selected extraction parameters (solvent [% of ethanol], temperature [°C], and time [min]) on DPPH free radical inhibition of two squash landraces (Bejaoui and Karkoubi) by-products (fibrous strands, peels, and seeds).

The variations in DPPH radical IPs were summarized in the heatmaps illustrated in Figure 1. Based on the data clustering, it can be suggested that the best maceration conditions to achieve the highest DPPH radical IP depend on several factors, including temperature, time, and ethanol percentage. In particular, the optimal extraction parameters were as follows: for the fibrous strands of Bejaoui, the optimal condition was 50% ethanol, with a temperature between 30 and 40°C, and an extraction time of 30 min. For Bejaoui peels, the best extraction conditions were 50% ethanol, with a temperature between 40 and 50°C, and an extraction time between 30 and 60 min. Conversely, the optimal conditions for the seeds of Bejaoui landrace were 30% ethanol, with a temperature between 30 and 40°C, and an extraction time between 5 and 15 min, while for Karkoubi fibrous strands, the optimal extraction conditions were 30% ethanol, a temperature of 40°C, and an extraction time of 15 min. In addition, for Karkoubi peels, the optimal conditions were 10% ethanol, a temperature of around 40°C, and an extraction time of 30 min. Finally, for Karkoubi seeds, the conditions were 10% ethanol, at a temperature around 50–60°C, and between 15 and 30 min of extraction time. These results confirm the importance of carefully choosing the extraction parameters and the necessity of thorough optimization to obtain the desired bioactive compounds.

### 3.2 Optimization of the antioxidant activity by using RSM

#### 3.2.1 Experimental domains

The experimental design was applied to the tested squash by-products, peels, fibrous strands, and seeds of both landraces (Bejaoui and Karkoubi). The central values and variation steps of the three factors (ethanol percentage [%], temperature [°C], and time [min]) for each type of by-product were deduced from the preliminary assessment for maceration processing, as illustrated in Table 2.

**Table 2:** Central values and variation steps of extraction parameters for the tested squash by-products (peels, fibrous strands, and seeds) of two squash landraces (Bejaoui and Karkoubi)

Factor	Center	Variation step	Center	Variation step	Center	Variation step
	Peels		Fibrous strands		Seeds	
**Bejaoui**						
Ethanol (%)	50	5	50	5	30	5
Temperature (°C)	40	5	35	5	35	5
Time (min)	45	15	30	5	22.5	7.5
**Karkoubi**						
Ethanol (%)	15	5	30	10	10	5
Temperature (°C)	45	5	45	5	55	5
Time (min)	45	15	10	5	22.5	7.5

#### 3.2.2 Experimental design and results of maceration extraction of squash by-products for TPC and DPPH antioxidant activity

Table 3 presents the experimental design matrix for the maceration extraction of squash by-products, with 17 experiments conducted on three different by-products (peels, fibrous strands, and seeds) of the studied landraces (Bejaoui and Karkoubi). The results were expressed in mg GAE/g DW for TPC and in DPPH percent inhibition for the antioxidant activity. According to Table 2, the highest TPC responses were observed in the fibrous strands of Bejaoui in experiments 13–17, with the content ranging between 74.34 and 75.81 mg GAE/g DW. The same experiments resulted in the highest TPC for the other by-products of the Bejaoui landrace, as well as for all the tested by-products of the Karkoubi landrace. Similar results were recorded for the antioxidant activity, where the highest IP was recorded for experiments 13–17 for all the by-products and both landraces, except for Karkoubi fibrous strands, where the most efficient experiments were 14–17. The highest IP was measured in the seed extracts of both landraces (up to 89.63 and 84.78% for Bejaoui and Karkoubi seeds, respectively), followed by fibrous strands and peels in the case of Karkoubi, or peels and fibrous strands in Bejaoui extracts.

**Table 3:** Experimental design matrix for maceration extraction of the tested by-products (peels, fibrous strands, and seeds) of two squash landraces (Bejoui and Karkoubi)

	Bejaoui						Karkoubi					
	Peels		Fibrous strands		Seeds		Peels		Fibrous strands		Seeds	
Exp *N*°	TPC	DPPH	TPC	DPPH	TPC	DPPH	TPC	DPPH	TPC	DPPH	TPC	DPPH
1	19.52	20.10	66.36	10.96	13.05	81.45	13.20	46.42	14.23	65.39	9.91	42.15
2	22.52	32.10	67.20	14.10	15.05	83.47	14.12	56.06	15.24	66.73	10.66	49.72
3	22.16	32.60	66.58	12.26	14.8	82.66	14.85	53.52	16.03	65.61	10.08	37.3
4	20.50	30.20	71.11	15.74	16.1	84.83	14.11	51.67	15.23	70.68	10.25	57.29
5	19.76	25.50	62.87	12.64	11.25	76.68	13.83	52.02	14.92	66.06	9.88	30.68
6	18.12	19.78	66.36	13.51	12.9	81.76	15.21	57.52	16.42	66.84	9.06	33.73
7	18.26	19.97	69.98	12.15	15.85	85.68	15.05	55.52	16.25	63.16	9.13	29.91
8	21.56	33.30	71.79	16.61	16.15	86.31	14.48	57.62	15.63	68.01	11.28	57.26
9	17.92	19.60	62.25	12.46	10.8	78.99	14.93	54.01	16.23	66.64	10.61	37.27
10	20.26	30.10	68.15	12.71	13.05	81.76	14.56	56.61	15.82	68.83	10.13	39.21
11	21.02	32.10	71.38	12.95	16.05	86.15	14.56	58.85	15.83	68.43	10.61	52.02
12	21.06	32.60	70.35	15.30	16.3	86.75	15.48	60.38	16.82	69.70	10.53	50.7
13	25.14	38.01	75.81	19.12	17.25	89.17	16.30	62.38	17.72	67.54	12.67	83.55
14	25.16	37.10	75.07	18.52	17.8	89.63	16.49	63.47	17.92	72.80	12.93	84.13
15	25.28	37.20	74.34	19.20	17.6	88.47	16.40	62.38	17.82	72.69	12.89	84.78
16	25.36	37.41	75.81	19.05	17.7	88.69	16.66	62.45	18.10	73.78	12.88	83.5
17	25.58	38.49	75.37	18.56	17.7	89.31	15.39	62.48	17.82	73.66	12.79	83.54

Different values of each variable have been enumerated in Table 1. TPC: Total phenolic compounds content expressed as mg GAE/g DW; DPPH: antioxidant activity expressed as IP (%).

#### 3.2.3 Regression and multivariate analysis

The provided ANOVA table (Table 3) displays the results of the statistical analysis conducted on the experimental design for the maceration extraction parameters of all the squash by-products. Results show the *F*-statistic and the level of significance (*p*-value) for each source of variation and each response variable. For the response variable of DPPH free radical scavenging activity, the highest *F*-statistic values for Bejaoui were observed for fibrous strands (136.703), followed by seeds (122.000) and peels (72.212), while for Karkoubi the highest *F*-statistic values were recorded for seeds (1259.842), followed by peels (65.377) and fibrous strands (39.562). For the response variable of TPC, the highest *F*-statistic values were observed for Bejaoui fibrous strands (155.918) and Bejaoui seeds (135.291), followed by Karkoubi seeds (103.004) and Bejaoui peels (62.409). These *F*-statistic values indicate that the source of variation also had a significant effect on the TPC response variable. The level of significance (*p*-value) for each source of variation and each response variable is also displayed in Table 4. The *p*-values for all the *F*-statistics were below the significance level of 0.05, indicating that the observed effects were statistically significant.

**Table 4:** ANOVA results for the extraction of TPC and DPPH free radical scavenging activity in by-products (peels, fibrous strands, and seeds) of two squash landraces (Bejoui and Karkoubi) via maceration

Responses	Source of variation	*F*-statistic	Significance	*F*-statistic	Significance
		Bejaoui		Karkoubi	
		Peels		Peels	
DPPH free radical scavenging activity	Regression	72.212	***	65.377	***
	Validity	6.351	ns (5.5%)	5.160	ns (7.5%)
	*R* ^2^	0.989		0.988	
	*R* ^2^_Adj_	0.976		0.973	
TPC	Regression	62.4092	***	9.692	**
	Validity	0.8807	ns (52.3%)	0.278	ns (83.9%)
	*R* ^2^	0.988		0.926	
	*R* ^2^_Adj_	0.972		0.830	
		**Fibrous strands**		**Fibrous strands**	
DPPH free radical scavenging activity	Regression	136.703	***	39.562	***
	Validity	0.226	ns (87.4%)	1.0391	ns (46.6%)
	*R* ^2^	0.994		0.981	
	*R* ^2^_Adj_	0.987		0.956	
TPC	Regression	155.918	***	52.395	***
	Validity	0.792	ns (55.9%)	3.843	ns (11.4%)
	*R* ^2^	0.994		0.985	
	*R* ^2^_Adj_	0.985		0.967	
		**Seeds**		**Seeds**	
DPPH free radical scavenging activity	Regression	122.000	***	1259.842	***
	Validity	0.934	ns (50.3%)	3.300	ns (14.0%)
	*R* ^2^	0.994		0.974	
	*R* ^2^_Adj_	0.986		0.924	
TPC	Regression	135.291	***	103.004	***
	Validity	2.138	ns (23.8%)	5.451	ns (6.9%)
	*R* ^2^	0.994		0.993	
	*R* ^2^_Adj_	0.987		0.983	

Significance: *p* < 0.01 (**) and *p* < 0.001 (***) mean very significant and strongly significant, respectively; ns: non-significant.

In the ANOVA table provided (Table 4), the *R* ^2^ values for all the regression models were high, ranging from 0.926 to 0.993, indicating that the independent variables explain a high percentage of the variability in the dependent variable.

In Table 4, the *R* ^2^_Adj_ values are slightly lower than the *R* ^2^ values, ranging from 0.830 to 0.987, which indicate that the regression models in our study provide a good fit to the data and suggest that the independent variables used in the experiments have a strong influence on the dependent variables.

#### 3.2.4 Effects of the extraction factors

Based on the experimental design methodology, the effect of the main factors on the DPPH free radical scavenging activity and TPC of the by-products (peels, fibrous strands, and seeds) of the studied squash landraces (Bejaoui and Karkoubi) was determined. Table 5 displays the significant factors and their corresponding coefficients for each by-product. For Bejaoui and Karkoubi peels, the linear effects of *b*_1_ and *b*_3_ had a highly significant influence (*p* < 0.01) on both DPPH and TPC. Additionally, the quadratic effect of *b*_11_ and *b*_22_ had a highly significant influence (*p* < 0.01) on both DPPH and TPC. For Bejaoui and Karkoubi fibrous strands, the linear effect of *b*_1_ had a highly significant influence (*p* < 0.01) on the DPPH scavenging activity, while the quadratic effects of *b*_11_ and *b*_22_ had a highly significant influence (*p* < 0.01) on both DPPH free radical scavenging activity and TPC. For Bejaoui and Karkoubi seeds, the linear effects of *b*_1_, *b*_2_, and *b*_3_ had a highly significant influence (*p* < 0.01) on both responses. Additionally, the quadratic effects of *b*_11_, *b*_22_, and *b*_33_ had a highly significant influence (*p* < 0.01) on both DPPH scavenging activity and TPC. Therefore, based on the results of the experimental design methodology, the highly significant factors for enhancing antioxidant and phenolic compounds from the squash by-products are the linear effects of *b*_1_, *b*_2_, and *b*_3_, as well as the quadratic effects of *b*_11_, *b*_22_, and *b*_33_.

**Table 5:** Coefficient significance concerning three extraction variables (solvent concentration [%], extraction temperature [°C] and extraction time [min]) and two responses (DPPH activity and TPC) of by-products (peels, fibrous strands, and seeds) of two squash landraces (Bejoui and Karkoubi)

	DPPH activity		TPC		DPPH activity		TPC	
	Coefficient	Signif.	Coefficient	Signif.	Coefficient	Signif.	Coefficient	Signif.
	Bejaoui peels				Karkoubi peels			
*b*_0_	37.642	***	25.104	***	83.900	***	12.832	***
**Linear effect**								
*b*_1_	2.151	***	0.500	***	7.244	***	0.281	ns (42.4%)
*b*_2_	2.700	***	0.338	***	0.418	*	0.100	ns (10.0%)
*b*_3_	2.874	***	0.817	***	6.125	**	0.234	ns (39.8%)
**Quadratic effect**								
*b*_11_	−6.427	***	−2.197	***	−22.097	***	−1.620	***
*b*_22_	−2.465	***	−1.732	***	−15.190	***	−0.987	**
*b*_33_	−6.577	**	−3.232	**	−23.909	10.2%	−1.375	ns (8.6%)
**Interaction effect**								
*b*_12_	−3.600	***	−1.165	***	3.107	***	−0.145	ns (8.1%)
*b*_13_	4.762	***	1.485	***	6.075	6.5%	0.742	*
*b*_23_	−2.500	**	−0.500	**	−0.812	51.7%	0.100	ns (15.9%)
	**Bejaoui Strands**				**Karkoubi Strands**			
*b*_0_	75.278	***	18.890	***	73.493	***	17.878	***
**Linear effect**								
*b*_1_	1.332	***	1.494	***	1.154	**	0.135	ns (11.7%)
*b*_2_	1.127	***	0.693	***	1.916	***	0.297	**
*b*_3_	2.983	***	0.712	***	2.430	***	0.143	ns (10.0%)
**Quadratic effect**								
*b*_11_	−3.874	***	−2.625	***	−3.449	***	−1.533	***
*b*_22_	−3.590	***	−2.999	***	−4.442	***	−1.162	***
*b*_33_	−3.655	***	−2.537	***	−3.726	***	−0.539	**
**Interaction effect**								
*b*_12_	0.925	ns (60.4%)	0.085	**	0.432	ns (38.7%)	−0.451	**
*b*_13_	−0.418	***	0.899	ns (13.5%)	−1.183	*	−0.529	**
*b*_23_	−1.731	*	0.525	***	0.595	ns (24.1%)	0.349	*
	**Bejaoui seeds**				**Karkoubi seeds**			
*b*_0_	89.053	***	3.522	***	83.900	***	12.832	***
**Linear effect**								
*b*_1_	1.240	***	0.131	***	7.244	***	0.281	***
*b*_2_	0.743	***	0.133	***	0.418	***	0.100	***
*b*_3_	3.211	***	0.409	***	6.125	***	0.234	***
**Quadratic effect**								
*b*_11_	−3.378	***	−0.287	***	−22.097	***	−1.620	***
*b*_22_	−2.571	***	−0.285	***	−15.190	***	−0.987	***
*b*_33_	−3.069	***	−0.427	***	−23.909	***	−1.375	***
**Interaction effect**								
*b*_12_	0.037	ns (87.2%)	−0.035	ns (21.9%)	3.107	***	−0.145	ns (14.4%)
*b*_13_	−1.114	*	−0.068	*	6.075	***	0.742	***
*b*_23_	−0.544	**	−0.100	ns (81.5%)	−0.812	ns (7.5%)	0.100	ns (29.7%)

Significance: *p* < 0.01 (**) and *p* < 0.001 (***) mean very significant and strongly significant, respectively; ns: non-significant.

To find the polynomial equation for each response, we used the provided equations and replaced the coefficients for each response using only significant coefficients, based on equation (1). The coefficients with a *p*-value greater than 0.05 were not considered significant and were excluded from the equation summarized in Table 6.

**Table 6:** Polynomial regression equations for predicting each response variable

Squash landraces	By-product	Response	Equation
Bejaoui	Peels	DPPH scavenging activity	*Y* = 37.642 + 2.151 × *X*_1_ + 2.700 × *X*_2_ + 2.874 × *X*_3_ − 6.427 × (*X*_1_ × *X*_1_) − 2.465 × (*X*_2_ × *X*_2_) − 6.577 × (*X*_3_ × *X*_3_) − 3.600 × (*X*_1_ × *X*_2_) + 4.762 × (*X*_1_ × *X*_3_) − 2.500 × (*X*_2_ × *X*_3_)
		TPC	*Y* = 25.104 + 0.500 × *X*_1_ + 0.338 × *X*_2_ + 0.817 × *X*_3_ − 2.197 × (*X*_1_ × *X*_1_) − 1.732 × (*X*_2_ × *X*_2_) − 3.232 × (*X*_3_ × *X*_3_) − 1.165 × (*X*_1_ × *X*_2_) + 1.485 × (*X*_1_ × *X*_3_) − 0.500 × (*X*_2_ ×*X*_3_)
	Fibrous strands	DPPH scavenging activity	*Y* = 75.278 − 3.874 × (*X*_1_ × *X*_1_) − 3.590 × (*X*_2_ × *X*_2_) − 3.655 × (*X*_3_ × *X*_3_) + 1.332 ×*X*_1_ + 1.127 × *X*_2_ + 2.983 × *X*_3_ + 0.925 × (*X*_1_ × *X*_2_) − 0.418 × (*X*_1_ × *X*_3_) − 1.731 × (*X*_2_ × *X*_3_)
		TPC	*Y* = 18.890 + 1.494 × *X*_1_ + 0.693 × *X*_2_ + 0.712 × *X*_3_ − 2.625 × (*X*_1_ × *X*_1_) − 2.999 × (*X*_2_ × *X*_2_) − 2.537 × (*X*_3_ × *X*_3_) + 0.085 × (*X*_1_ × *X*_2_) + 0.899 × (*X*_1_ × *X*_3_) + 0.525 × (*X*_2_ × *X*_3_)
	Seeds	DPPH scavenging activity	*Y* = 89.053 + 1.240 × *X*_1_ + 0.743 × *X*_2_ + 3.211 × *X*_3_ − 3.378 × (*X*_1_ × *X*_1_) − 2.571 × (*X*_2_ × *X*_2_) − 3.069 × (*X*_3_ × *X*_3_) + 0.037 × (*X*_1_ × *X*_2_) − 1.114 × (*X*_1_ × *X*_3_) − 0.544 × (*X*_2_ × *X*_3_)
		TPC	*Y* = 3.522 + 0.131 × *X*_1_ + 0.133 × *X*_2_ + 0.409 × *X*_3_ − 0.287 × (*X*_1_ × *X*_1_) − 0.285 × (*X*_2_ × *X*_2_) − 0.427 × (*X*_3_ × *X*_3_) − 0.068 × (*X*_1_ × *X*_3_) − 0.100 × (*X*_2_ × *X*_3_)
Karkoubi	Peels	DPPH scavenging activity	*Y* = 83.900 + 7.244 × *X*_1_ + 0.418 × *X*_2_ + 6.125 × *X*_3_ − 22.097 × (*X*_1_ × *X*_1_) − 15.190 × (*X*_2_ × *X*_2_) − 23.909 × (*X*_3_ × *X*_3_) + 3.107 × (*X*_1_ × *X*_2_) + 6.075 × (*X*_1_ × *X*_3_) − 0.812 × (*X*_2_ × *X*_3_)
		TPC	*Y* = 12.832 + 0.281 × *X*_1_ + 0.100 × *X*_2_ + 0.234 × *X*_3_ − 1.620 × (*X*_1_ × *X*_1_) − 0.987 × (*X*_2_ × *X*_2_) − 1.375 × (*X*_3_ × *X*_3_) − 0.145 × (*X*_1_ × *X*_2_) + 0.742 × (*X*_1_ × *X*_3_) + 0.100 × (*X*_2_ × *X*_3_)
	Fibrous strands	DPPH scavenging activity	*Y* = 73.493 − 3.449 × (*X*_1_ × *X*_1_) − 4.442 × (*X*_2_ × *X*_2_) − 3.726 × (*X*_3_ × *X*_3_) + 1.154 × *X*_1_ + 1.916 × *X*_2_ + 2.430 × *X*_3_ + 0.432 × (*X*_1_ × *X*_2_) − 1.183 × (*X*_1_ × *X*_3_) + 0.595 × (*X*_2_ × *X*_3_)
		TPC	*Y* = 17.878 + 0.135 × *X*_1_ + 0.297 × *X*_2_ + 0.143 × *X*_3_ − 1.533 × (*X*_1_ × *X*_1_) − 1.162 × (*X*_2_ × *X*_2_) − 0.539 × (*X*_3_ × *X*_3_) − 0.451 × (*X*_1_ × *X*_2_) − 0.529 × (*X*_1_ × *X*_3_) + 0.349 × (*X*_2_ × *X*_3_)
	Seeds	DPPH scavenging activity	*Y* = 83.900 + 7.244 × *X*_1_ + 0.418 × *X*_2_ + 6.125 × *X*_3_ − 22.097 × (*X*_1_ × *X*_1_) − 15.190 × (*X*_2_ × *X*_2_) − 23.909 × (*X*_3_ × *X*_3_) + 3.107 × (*X*_1_ × *X*_2_) + 6.075 × (*X*_1_ × *X*_3_) − 0.812 × (*X*_2_ × *X*_3_)
		TPC	*Y* = 12.832 + 0.281 × *X*_1_ + 0.100 × *X*_2_ + 0.234 × *X*_3_ − 1.620 × (*X*_1_ × *X*_1_) − 0.987 × (*X*_2_ × *X*_2_) − 1.375 × (*X*_3_ × *X*_3_) + 0.742 × (*X*_1_ × *X*_3_) + 0.100 × (*X*_2_ × *X*_3_)

#### 3.2.5 Graphical study of response surfaces

**Figure 2:**
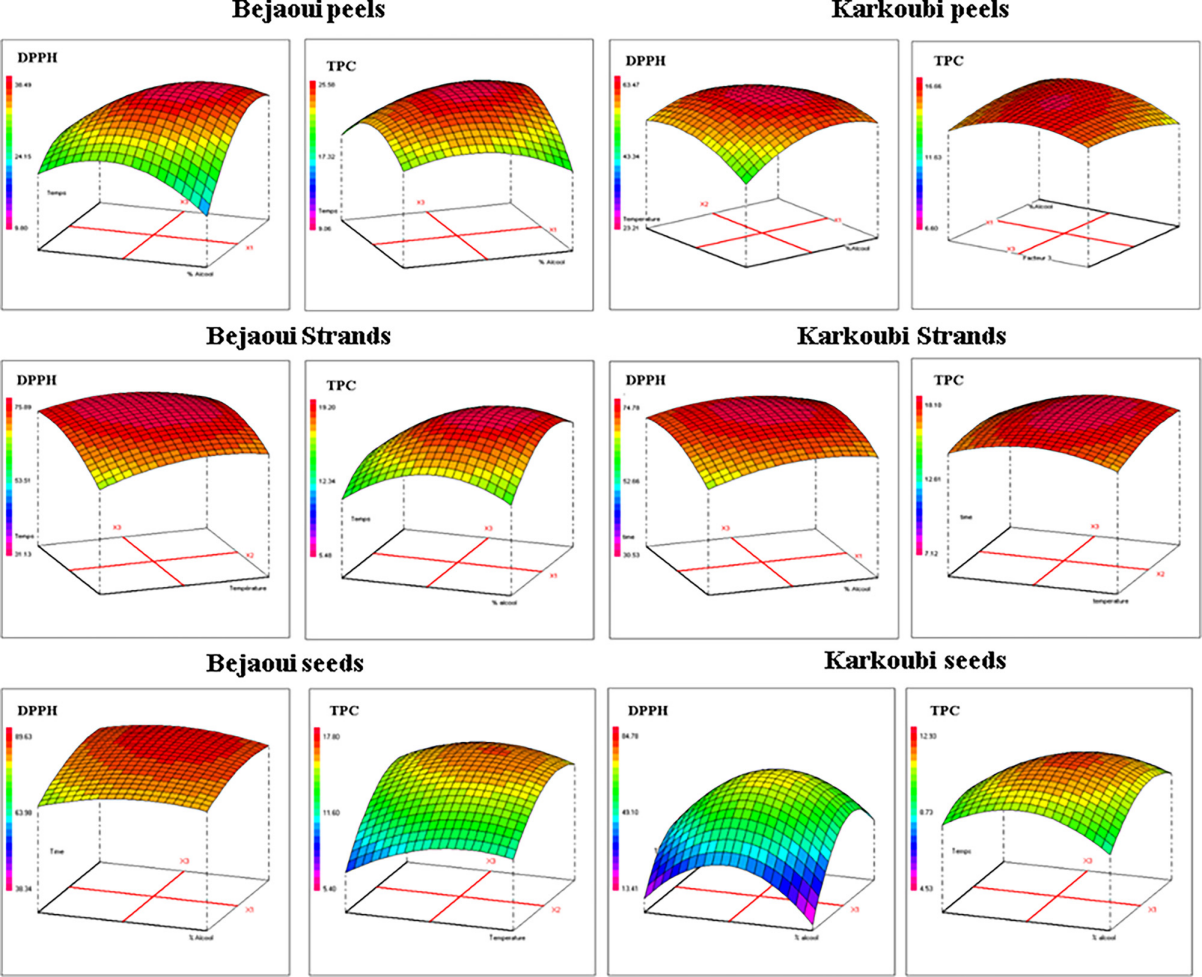
3D graphical study of the response surface optimization for DPPH inhibition assay and TPC of by-products (peels, fibrous strands, and seeds) of two squash landraces (Bejaoui and Karkoubi) via maceration extraction.

The results of the response surface analysis for squash by-products revealed that the interactions between the percentage of ethanol and the extraction time or temperature had a significant influence on the levels of DPPH inhibition (Figure 2). The highest inhibition of DPPH was recorded when the percentage of ethanol, the extraction duration, and extraction temperature were high for all the tested by-products. Regarding Bejaoui peels, the highest inhibition of DPPH was recorded when the percentage of ethanol and extraction time were combined, reaching an IP of 38%. For Karkoubi peels, the highest inhibition of DPPH was obtained by combining the percentage of ethanol and extraction temperature, reaching an IP of 64%. Results for Bejaoui and Karkoubi fibrous strands were similar, with the maximum IP of DPPH being recorded when the percentage of ethanol and the extraction time were combined, reaching 75 and 74% DPPH inhibition, respectively. The highest DPPH inhibition was recorded for Bejaoui and Karkoubi seeds, reaching an IP of 89 and 84%, respectively, when the percentage of ethanol and extraction time were combined. These results suggest that the percentage of ethanol and the extraction time or temperature were important factors for optimizing DPPH inhibition levels for the tested squash by-products.

Results of the response surface analysis for by-products of the studied landraces showed that the interactions between ethanol percentage, extraction time, and temperature also had a significant influence on total polyphenol contents (Figure 2). Regarding Bejaoui peels, the highest TPC values were obtained for the interaction of ethanol percentage and extraction time (values up to 25 mg GAE/g DW). Similar results were observed for Karkoubi peels and the fibrous strands of both landraces, where the highest TPC values were recorded for the interaction of ethanol percentage and extraction time or temperature and extraction time. However, although the highest TPC for Karkoubi seeds was obtained for the interaction of ethanol percentage and extraction time, the obtained values were relatively low compared to the other squash by-products. These results indicate that optimizing the extraction conditions is crucial for obtaining high TPC from different by-products of squash fruit.

#### 3.2.6 Results and analysis of desirability

The desirability table (Table 7) presented the optimization results of the extraction conditions for three squash by-products of two different landraces. It includes the responses (DPPH and TPC), their values, the percentage of their contribution (*D*%), and the factors (solvent percentage [%], extraction temperature [°C], and extraction time [min]) used for the optimization. Additionally, both the coded values and real values of the factors are provided.

**Table 7:** Desirability results and optimal extraction parameters (solvent concentration [%], extraction temperature [°C], and extraction time [min]) of by-products (peels, fibrous strands, and seeds) two squash landraces (Bejaoui and Karkoubi)

Factors	Optimal extraction parameters	Responses	Predicted values	Experimental values	*D* (%)
**Bejaoui peels**					
Ethanol %	51.54	DPPH	37.64	36.47 ± 1.02	100
Temperature	40.76	TPC	25.10	21.52 ± 0.77	100
Time	50.45	Total desirability			98.16
**Bejaoui fibrous strands**					
Ethanol %	50.38	DPPH	75.28	77.46 ± 3.41	100
Temperature	37.09	TPC	18.89	16.99 ± 1.15	100
Time	36.31	Total desirability			100
**Bejaoui seeds**					
Ethanol %	30.01	DPPH	17.61	18.88 ± 4.11	100
Temperature	36.36	TPC	89.05	99.74 ± 3.86	100
Time	7.91	Total desirability			100
**Karkoubi peels**					
Ethanol %	13.19	DPPH	62.63	62.71 ± 4.11	100
Temperature	43.43	TPC	16.25	14.14 ± 2.97	100
Time	47.18	Total desirability			100
**Karkoubi fibrous strands**					
Ethanol %	33.37	DPPH	73.49	74.88 ± 2.89	100
Temperature	46.65	TPC	17.88	17.14 ± 1.66	100
Time	10.82	Total desirability			100
**Karkoubi seeds**					
Ethanol %	10.65	DPPH	12.85	14.73 ± 4.93	100
Temperature	55.34	TPC	84.87	87.22 ± 4.78	100
Time	23.16	Total desirability			100

For Bejaoui peels, the highest desirability was achieved with a solvent percentage of 51.54%, extraction temperature of 40.76°C, and extraction time of 50.45 min (Table 7). Similarly, for Bejaoui fibrous strands, the highest desirability was obtained with a solvent percentage of 50.38%, an extraction temperature of 37.09°C, and an extraction time of 36.31 min, while for Bejaoui seeds, the highest desirability was obtained with a solvent percentage of 30.01%, an extraction temperature of 36.36°C, and an extraction time of 7.91 min. For Karkoubi peels, fibrous strands, and seeds, the highest desirability was also achieved with different combinations of the three factors. Furthermore, the seeds of both squash landraces were analyzed for their TPC, which varied between 84.87 and 89.05 mg GAE/g of extracts. Remarkably, for all by-products, the predicted values of DPPH and TPC exhibited remarkable similarity with their respective experimental values. These results indicate a close correlation between the DPPH and TPC values across all the studied extraction points, suggesting consistent antioxidant activity and phenolic content in squash by-products.

Overall, the results suggest that the optimization of the extraction process could lead to higher yields of antioxidants from squash by-products, which could have potential applications in the food and nutraceutical industries as novel natural antimicrobial agents.

### 3.3 Quantification of phenolic compounds in squash by-products using HPLC

Table 8 displays the compositions of phenolic compounds in the studied by-products of Bejaoui and Karkoubi landraces, as identified through HPLC analysis. Our results showed distinct profiles for each landrace, with varying concentrations of phenolic compounds across different parts of the squash fruit. Twelve phenolic compounds were detected in all the studied by-products of both landraces. Notably, the epigallocatechin content was significantly high in the fibrous strands of both landraces (24.41 mg/g DE and 21.88 mg/g DE for Bejaoui and Karkoubi landraces, respectively), followed by peels (4.78 mg/g DE and 3.41 mg/g DE for Bejaoui and Karkoubi landraces, respectively) and seeds (0.17 mg/g DE and 0.19 mg/g DE for Bejaoui and Karkoubi landraces, respectively). Both landraces contained vanillic acid mostly in their fibrous strands (Bejaoui: 1.01 mg/g DE and Karkoubi: 0.75 mg/g DE), while lower contents were recorded in peels (Bejaoui: 0.25 mg/g DE, Karkoubi: 0.18 mg/g DE) and seeds (Bejaoui: 0.08 mg/g DE, Karkoubi: 0.05 mg/g DE). Gallic acid was also present in all by-products, with similar concentrations for both landraces. Catechin gallate was more abundant in Bejaoui seeds and peels (1.05 and 1.44 mg/g DE) than in Karkoubi, while Karkoubi had higher concentrations in the seeds (1.19 mg/g DE). Hydroxytyrosol was detected only in the peels and fibrous strands of both varieties (Bejaoui: 2.05 mg/g DE, Karkoubi: 2.95 mg/g DE for peels and Bejaoui: 0.98 mg/g DE, Karkoubi: 0.77 mg/g DE for fibrous strands). Chlorogenic acid was found in Bejaoui and Karkoubi seeds (0.47 and 0.55 mg/g DE, respectively) and fibrous strands (0.44 and 0.76 mg/g DE, respectively) but was not detected in the peels. Epicatechin was more abundant in fibrous strands (10.47 mg/g DE and 11.41 mg/g DE for Bejaoui and Karkoubi landraces, respectively), while quercetin was present in Bejaoui peels (0.05 mg/g DE) and fibrous strands (0.01 mg/g DE), as well as in Karkoubi peels (0.02 mg/g DE) and fibrous strands (0.01 mg/g DE). Rutin was detected in low amounts in all the studied by-products of both varieties, while ferulic acid was detected in similar amounts in the seeds of both landraces (0.01 mg/g DE). Myricetin was detected in Bejaoui peels and fibrous strands (0.05 mg/g DE and 0.03 mg/g DE, respectively), as well as in Karkoubi peels (0.05 mg/g DE). Finally, resveratrol was detected in seeds and peels of both landraces but not in the fibrous strands. These findings emphasized the varied composition of phenolic compounds depending on the landrace and the tested by-product, indicating potential applications in the food and health industries through their bioactivities.

**Table 8:** Phenolic compounds content in the extracts of the studied by-products of two squash landraces (Bejaoui and Karkoubi) (contents are expressed as mg/g DE)

Identification	Bejaoui			Karkoubi		
	Seeds	Peels	Fibrous strands	Seeds	Peels	Fibrous strands
Vanillic acid	0.08 ± 0.02^c^	0.25 ± 0.09^b^	1.01 ± 0.01^a^	0.05 ± 0.02^c^	0.18 ± 0.09^b^	0.75 ± 0.01^a^
Gallic acid	0.08 ± 0.01^a^	0.01 ± 0.01^c^	0.03 ± 0.01^b^	0.05 ± 0.01^a^	0.01 ± 0.00^b^	0.01 ± 0.00^b^
Catechin gallate	1.05 ± 0.22^a^	1.44 ± 0.41^a^	0.08 ± 0.01^b^	1.19 ± 0.18^a^	0.69 ± 0.03^b^	0.01 ± 0.03^c^
Hydroxytyrosol	0	2.05 ± 0.34^a^	0.98 ± 0.11^b^	0	2.95 ± 0.18^a^	0.77 ± 0.01^b^
Epigallocatechin	0.17 ± 0.02^c^	4.78 ± 0.52^b^	24.41 ± 2.07^a^	0.19 ± 0.04^c^	3.41 ± 0.40^b^	21.88 ± 2.75^a^
Chlorogenic acid	0.47 ± 0.01^a^	0	0.44 ± 0.01^a^	0.55 ± 0.01^b^	0	0.76 ± 0.03^a^
Epicatechin	3.11 ± 0.24^b^	1.43 ± 0.44^c^	10.47 ± 0.75^a^	2.78 ± 0.15^c^	4.27 ± 0.51^b^	11.41 ± 0.75^a^
Quercitin	0	0.05 ± 0.01^a^	0.01 ± 0.00^b^	0	0.02 ± 0.01^a^	0.01 ± 0.01^b^
Rutin	0.75 ± 0.12^a^	0.14 ± 0.03^b^	0.11 ± 0.01^b^	0.72 ± 0.04^a^	0.21 ± 0.03^c^	0.51 ± 0.04^b^
Ferulic acid	0.01 ± 0.00	0	0	0.01 ± 0.00	0	0
Myrecitin	0	0.05 ± 0.01^a^	0.03 ± 0.01^b^	0	0.05 ± 0.02	0
Resveratrol	0.45 ± 0.08^b^	0.75 ± 0.01^a^	0	0.59 ± 0.08^b^	0.78 ± 0.08^a^	0

Values are expressed as means of triplicates ± standard deviation. For each variety, values with different superscripts within the same row are significantly different at *p* < 0.05, according to Duncan’s multiple range test.

### 3.4 Antibacterial activity of different squash by-products

The antibacterial activities of squash by-products against five bacterial strains are presented in Table 9. Generally, the extracts of the studied by-products inhibited significantly the growth of the two Gram-positive (*E. faecalis* and *B. subtilis*) and the three Gram-negative bacteria (*S. enterica*, *P. aeruginosa,* and *E. coli*) at different extent. The presented results outline the antibacterial activity by quantifying the percentage of bacterial growth inhibition, based on equation (2). In general, the extracts of Bejaoui seeds could be suggested as potent antibacterial agents, showcasing impressive effectiveness with IPs ranging from 59 to 78% across all the bacterial strains. Similar results were recorded for the extracts of Karkoubi seeds, although the obtained IPs were lower than the respective ones of Bejaoui seeds. The fibrous strands of Bejaoui fruit also exhibited considerable antibacterial activity for all the tested bacterial strains except for the case of *S. enterica* where lower activity was recorded. In the case of peel extracts of Bejaoui fruit, significant activity was recorded only against *E. faecalis* and *P. aeruginosa*. Conversely, Karkoubi peels and fibrous strands did not show significant antibacterial effects against any of the studied bacterial strains.

**Table 9:** Antibacterial activity (% of growth inhibition) of the optimized extracts of by-products (peels, fibrous strands, and seeds) of two squash landraces (Bejoui and Karkoubi)

		*S. enterica*	*E. faecalis*	*P. aeruginosa*	*E. coli*	*B. subtilis*
Bejaoui	Peels	2.37^Ee^ ± 0.19	21.13^Bb^ ± 12.53	45.67^Ca^ ± 14.38	6.73^Cc^ ± 2.40	5.77^Dd^ ± 1.26
	Fibrous strands	18.64^Cd^ ± 6.57	51.89^Ab^ ± 0.03	60.23^Ba^ ± 3.93	25.57^Bc^ ± 8.10	25.78^Cc^ ± 6.35
	Seeds	62.41^Abc^ ± 4.8	58.68^Ac^ ± 8.39	78.03^Aa^ ± 4.32	68.41^Ab^ ± 4.49	65.68^Abc^ ± 5.35
Karkoubi	Peels	21.51^Ca^ ± 11.21	16.98^Bb^ ± 4.95	5.75^Ec^ ± 0.99	25.94^Ba^ ± 4.49	20.35^Ca^ ± 7.9
	Fibrous strands	8.29^Db^ ± 3.34	14.52^Ba^ ± 3.87	16.43^Da^ ± 5.24	1.22^Dc^ ± 1.34	9.96^Db^ ± 4.38
	Seeds	52.31^Bb^ ± 4.03	56.33^Ab^ ± 5.42	62.4^Ba^ ± 5.56	62.02^Aa^ ± 15.5	49.15^Bb^ ± 12.79

Values are expressed as means of six replicates (hexaplicates) ± standard deviation. Values with different uppercase superscripts indicate significant differences between the various pumpkin by-products, while values with different lowercase superscripts denote significant differences between bacterial strains. Statistical significance was determined at *p* < 0.05 using Duncan’s multiple range test.

## 4 Discussion

The extraction of phytochemical antioxidants from plant sources has gained significant attention in recent years due to their potential health-promoting properties and the consumers’ awareness for the use of synthetic compounds in food products [22]. These bioactive compounds, such as phenolic compounds and flavonoids, possess remarkable antioxidant capabilities that can neutralize harmful free radicals and protect against oxidative stress-related diseases [22]. To maximize the extraction efficiency of these valuable antioxidants, researchers have been exploring innovative approaches to intensify the extraction process and optimize the yields of bioactive compounds obtained from plant matrices. For this purpose, the optimization of various extraction parameters, such as the solvent type, solvent concentration, temperature, and extraction time, has been investigated aiming to enhance the efficiency and effectiveness of the extraction process [12,23]. The findings of the present study regarding the optimal extraction parameters for obtaining extracts with high antioxidant activity from different squash by-products were in line with previous literature reports on the antioxidant activity of squash. In particular, several studies have investigated the effect of extraction parameters on the antioxidant properties of squash by-products, including the fibrous strands, peels, and seeds [24,25]. Moreover, Singh et al. [26] reported that the extracts of squash peels showed higher antioxidant activity when extracted with 50% ethanol at 25°C. It is also worth noting that the antioxidant activity of squash by-products may be influenced by various pre- and post-harvest factors, including the squash genotype, the processing conditions, as well as the analytical methods used [27–29]. In order to achieve precise and efficient extraction, the use of mathematical modeling, particularly RSM, has been widely implemented as an efficient and practical tool [30]. This method enables the systematic exploration of multiple extraction parameters to identify optimal conditions for maximizing the yield of antioxidants from plants. This approach not only aids in better understanding the complex interactions between extraction parameters but also helps to reduce the consumption of solvents and energy, making the extraction process more sustainable and economically viable [30].

In our work, it was found that in the Bejaoui landrace, the experimental values of DPPH antioxidant activity and TPC varied significantly among the different by-products (e.g., peels, fibrous strands, and seeds). The recorded high *F*-statistic values suggest that the source of variation (i.e., the different types of by-products) had a significant effect on the DPPH response and TPC variable. The seeds exhibited the highest TPC (99.74 mg GAE/g DE; Table 7), indicating a rich source of phenolic compounds, while the fibrous strands displayed the highest DPPH antioxidant activity (78%; Table 7), highlighting their strong free radical scavenging ability and also suggesting that other compounds except for phenolic compounds may be responsible for the antioxidant activity of this particular plant matrix. The Karkoubi landrace showed similar trends, where the seeds recorded a high TPC value (87.22 mg GAE/g DE), while the fibrous strands demonstrated a strong antioxidant activity of 75%. In a similar study, Ben Mansour et al. [12] evaluated the antioxidant properties of different parts of the Tunisian squash landrace Batati using extraction conditions comparable to the present study. The authors also suggested a varied response of the extraction efficiency depending on the various parameters of the extraction protocol (e.g., solvent concentration [%], extraction temperature [°C], and extraction time [min]), while the optimal extraction conditions differed among the studied by-products [12]. Compared to our results, we observed similar trends in DPPH scavenging activity and TPC for the different parts of the squash, with values closely matching those reported by Ben Mansour et al. [12]. Similarly, Singh et al. [26] reported a limited DPPH free radical scavenging activity of 44% for 70% methanolic extracts of squash peel, while Asif et al. [31] found that squash peel extracts exhibited antioxidant activity of only 69% when using 65, 80, and 99.9% methanolic extracts. Moreover, Kulczyński et al. [25] reported lower inhibition values of 20.5 and 18.9% for squash seed extracts using 70% ethanol and 70% methanol, respectively. In contrast, our results revealed considerably higher DPPH antioxidant activity percentages in seed extracts of both Bejaoui and Karkoubi landraces (up to 89.63 and 84.78%, respectively), depending on the extraction conditions. Additionally, the obtained TPC values of seeds also exhibited a wide range (from 10.8 to 17.8 mg GAE/g DE in the case of Bejaoui landrace and from 9.06 to 12.93 mg GAE/g DE, in the case of Karkoubi landrace). On the other hand, the highest TPC values were recorded in the fibrous strands of Bejaoui landrace (up to 75.81 mg GAE/g DE), indicating that this particular by-product is a rich source of phenolic compounds. These variations in antioxidant activity and TPC highlight the influence of squash genotype, the conditions of the extraction method, and the fruit part on the bioactive properties of the squash by-products [25,30,32]. Therefore, it is crucial to consider these factors for potential applications of squash by-products in the food and nutraceutical industries and the development of functional foods aiming to obtain extracts with improved antioxidant activity and associated health benefits [33].

Apart from promising antioxidant activity, the extracts showed significant antibacterial activity, consistent with the reports of previous studies. Asif et al. [31] studied the antibacterial activity of methanolic extracts of squash peels against four bacterial strains including *E. coli*, *P. multocida*, *S. aureus,* and *B. subtilis* and reported interesting antibacterial potential. In a research undertaken by Chonoko and Rufai [34], the phytochemical screening and antimicrobial potential of squash revealed substantial antibacterial properties for the ethanolic and methanolic extracts derived from squash peels. Remarkably, these extracts demonstrated pronounced antibacterial efficacy against the *S. aureus* and *S. typhi* bacterial strains. Similarly, Hussain et al. [35] reported that squash fibrous strands extract exhibited greater inhibition against *E. coli* and *B. subtilis* than peels and seeds extracts. Moreover, Badr et al. [36] determined the chemical composition and biological activity of ripe squash fruit parts and suggested that squash rind and strand extracts exhibited moderate antimicrobial activities against the Gram-positive bacteria *B. subtilis* and *B. cereus* and considerable inhibition activity against the bacterial strain *S. viridochromogenes*. According to Caili et al. [37], the antibacterial activity of squash extracts was found to be related to bioactive compounds being present in fruit parts and seeds. Phytochemicals such as phenolic compounds, commonly present in squash by-products, could play an important role as antibacterial agents and are associated with the antimicrobial properties of the obtained extracts [34]. These properties of phenolic compounds have been associated with their reaction with cellular components, resulting in the leakage of nucleotides and proteinaceous material into extracellular areas [35].

The relationship between antioxidant and antibacterial activities can be closely linked to the phenolic composition of the Bejaoui and Karkoubi landraces. Phenolic compounds are well-known for their antioxidant properties, which enable them to neutralize free radicals and protect cells from oxidative stress [3]. As reported by Ben Mansour et al. [12], the presence of potent antioxidant compounds, such as vanillic acid, catechin gallate, and epigallocatechin, in specific parts of the squash fruit may contribute to their antioxidant activities [38]. Additionally, phenolic compounds such as resveratrol, epicatechin, and quercetin and their derivatives have been reported to possess antibacterial properties [39]. Moreover, hydroxytyrosol, which was detected mostly in the peels of the studied landraces (Bejaoui and Karkoubi), has been shown to exhibit significant antibacterial activity against certain pathogens [40,41]. Similarly, other phenolic compounds like epicatechin and chlorogenic acid may contribute to the antibacterial potential observed in different plant matrices [42]. The presence or absence of specific phenolic compounds in different parts of the squash varieties could influence their antibacterial activities, highlighting the importance of phenolic composition analysis in understanding the health-promoting properties of squash by-products and their potential as a natural source of antioxidants and antibacterial agents [12]. However, it should be highlighted that several phytochemicals may contribute to the overall bioactive capacity of pumpkin by-products (e.g. tocopherols, sterols, carotenoids, alkaloids, terpenoids, etc. [43–46]) that could explain the variability between the obtained TPC content and the antimicrobial properties detected in our study.

## 5 Conclusion

This study demonstrated that extraction conditions, as analyzed through RSM, play a critical role in influencing the TPC of squash by-products, offering valuable insights for optimizing extraction processes to maximize their bioactive potential. The HPLC analysis of the obtained extracts revealed major phenolic compounds, such as vanillic acid, catechin gallate, hydroxytyrosol, epigallocatechin, chlorogenic acid, and epicatechin, which contribute to the antioxidant and antibacterial properties of both studied landraces. Notably, the interaction between ethanol concentration, extraction time, and temperature significantly impacted the antioxidant capacity, underscoring the importance of fine-tuning these parameters to enhance the bioactivity of the extracts. This optimization process is crucial for leveraging squash by-products as a valuable, sustainable source of natural antioxidant and antimicrobial agents with potential applications in the food and nutraceutical industries. The findings support the integration of eco-friendly extraction techniques for the valorization of agricultural waste, contributing to both environmental sustainability and the development of health-promoting products. However, further studies are needed using more genotypes in order to reveal the genotypic variability of the species in terms of bioactive compound content, as well as to focus on the optimization of the extraction of other bioactive compounds such as carotenoids which also have health-beneficial properties and are abundant in *Cucurbita* species.
